# Multiplex RT-PCR Amplification of HIV Genes to Create a Completely Autologous DC-Based Immunotherapy for the Treatment of HIV Infection

**DOI:** 10.1371/journal.pone.0001489

**Published:** 2008-01-30

**Authors:** Irina Tcherepanova, Jason Harris, Aijing Starr, Jaclyn Cleveland, Helen Ketteringham, David Calderhead, Joe Horvatinovich, Don Healey, Charles A. Nicolette

**Affiliations:** Research and Development Department, Argos Therapeutics, Inc., Durham, North Carolina, United States of America; AIDS Research Center, Chinese Academy of Medical Sciences and Peking Union Medical College, China

## Abstract

**Background:**

Effective therapy for HIV-infected individuals remains an unmet medical need. Promising clinical trials with dendritic cell (DC)-based immunotherapy consisting of autologous DC loaded with autologous virus have been reported, however, these approaches depend on large numbers of HIV virions to generate sufficient doses for even limited treatment regimens.

**Methodology/Principal Findings:**

The present study describes a novel approach for RT-PCR amplification of HIV antigens. Previously, RT-PCR amplification of autologous viral sequences has been confounded by the high mutation rate of the virus which results in unreliable primer-template binding. To resolve this problem we developed a multiplex RT-PCR strategy that allows reliable strain-independent amplification of highly polymorphic target antigens from any patient and requires neither viral sequence data nor custom-designed PCR primers for each individual. We demonstrate the application of our RT-PCR process to amplify translationally-competent RNA encoding regions of Gag, Vpr, Rev and Nef. The products amplified using this method represent a complex mixture of autologous antigens encoded by viral quasispecies. We further demonstrate that DCs electroporated with *in vitro*-transcribed HIV RNAs are capable of stimulating poly-antigen-specific CD8+ T cell responses *in vitro*.

**Conclusion/Significance:**

This study describes a strategy to overcome patient to patient viral diversity enabling strain-independent RT-PCR amplification of RNAs encoding sequence divergent quasispecies of Gag, Vpr, Rev and Nef from small volumes of infectious plasma. The approach allows creation of a completely autologous therapy that does not require advance knowledge of the HIV genomic sequences, does not have yield limitations and has no intact virus in the final product. The simultaneous use of autologous viral antigens and DCs may provoke broad patient-specific immune responses that could potentially induce effective control of viral loads in the absence of conventional antiretroviral drug therapy.

## Introduction

Immunotherapeutic strategies for HIV-infected individuals are focused on eliciting antiviral CD8+ T cell responses to control the level of HIV virus *in vivo*. Evidence that cellular immune responses play an important role in controlling HIV infection is supported by several observations including: a) Frequencies of CTL inversely correlate with HIV plasma levels [1] b) Blocking CD8+ T cells with anti-CD8-specific antibodies in SIV-infected macaques correlates with loss of viral control [2], c) resolution of acute viremia in the SIV macaque model requires circulating CD8+ T [3], d) The presence of virus-specific CTL coincides with the appearance of mutant viruses which are no longer recognized by these CTL [4], and e) The presence of CD8+CD28+ T cells are reportedly associated with long-term non-progression [5], [6].

Many current HIV immunotherapies utilize individual consensus antigens or defined epitopes derived from those reference HIV sequences. Potential therapies based on clade-specific consensus antigens have been investigated in over 80 clinical trials, however, the results demonstrate a consistent lack of efficacy [7]–[9]. While augmentation of immune responses to consensus sequences used for immunization was demonstrated, these therapies did not result in reduction of viral loads. Evidence suggests that the lack of HIV-protective immunity is attributed to sequence divergence between autologous and consensus antigens. The high HIV mutation rate results in novel variants which encode point mutations within CTL epitopes and escape recognition by specific T cells. Studies with overlapping peptides confirmed that CTL recognizing autologous peptides encoded within a known HIV virus did not cross react with corresponding consensus sequences [10]. Studies on humans and non-human primates correlate virus escape from CTL with progression to AIDS [11]–[13]. In addition, each patient creates a unique environment for its own viral evolution. Consequently, there is substantial mutational variation between the virus infecting the patient and the reference sequences upon which most HIV immunotherapies are based. Also, since virus sequence diversity defines HIV clades, therapies based on consensus antigens from one clade may have limited ability to cross control evolutionally divergent viruses from other clades. Therefore, therapies based on autologous viral antigens would have broader applicability since the therapy would be perfectly matched to the virus species infecting each subject. To date, the only successful immunotherapies for HIV-infected patients used dendritic cells loaded with autologous viral antigens. Independent clinical studies by Lu et al., and Garcia et.al., demonstrated for the first time that immunization with inactivated whole autologous HIV virus-loaded DC therapy can lead to durable control of viral load [14], [15]. Although these clinical studies demonstrated the potential utility of an autologous DC therapy, the choice of whole inactivated HIV virus as an immunogen is not ideal and may have significant safety and practical limitations.

In the present study we report on strain-independent RT-PCR amplification of four HIV antigens to generate templates for *in vitro*-transcribed RNA. Previously a major obstacle to RT-PCR amplification of autologous viral sequences was designing functional PCR primers due to the high mutation rate of the virus. To resolve this problem, we developed a multiplex RT-PCR strategy that allows reliable strain-independent amplification of highly polymorphic target antigens from any patient without the requirement for first knowing the viral sequence and custom-designing of PCR primers for each individual. The amplified products contain a complex mixture of autologous antigens encoded by viral quasispecies. We further demonstrate that *in vitro*-transcribed RNA can be delivered to DCs where the encoded antigens are expressed, processed, and presented by MHC class I molecules on the cell surface.

Regions for Gag, Vpr, Rev and Nef were selected for amplification to generate this completely autologous RNA-transfected DC therapy which lacks infectious virus in the final formulation, thereby circumventing potential safety concerns. The choice of antigens targeted for amplification was based on the several criteria: a) Substantial regions of the target gene had to be amenable to PCR amplification using our primer design strategy, b) Antigen expression should not adversely affect dendritic cell function and c) Antigens had to induce functional CTLs [16]–[21]. The simultaneous use of autologous viral antigens and DCs may provide for a broad patient-specific immune response that could potentially provide better control of residual virus or rebound of virus following the cessation of antiretroviral therapy.

## Results

### Successful amplification of multiple HIV RNA antigens using the devised multiplex RT-PCR protocol

**Figure 1:**
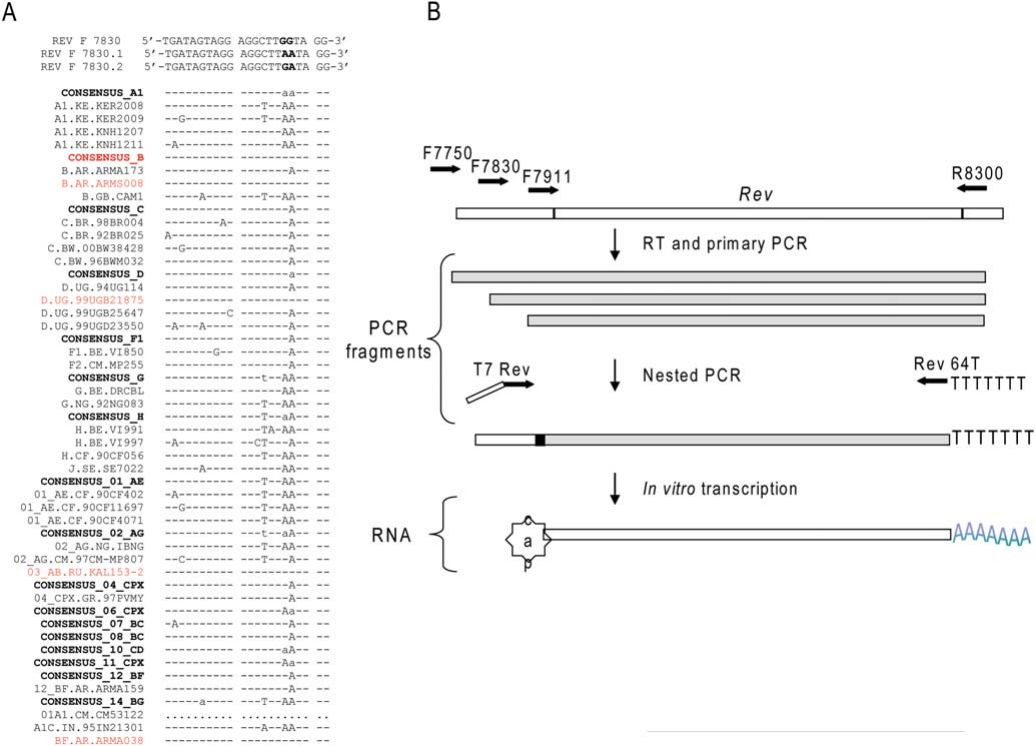
Amplification of autologous HIV sequences using multiplex PCR. Panel A. Sequence alignment of multiple HIV isolates, revealed a region of relative conservation with variable residues in positions 7847 and 7848. Primer REVF7830 is perfectly complimentary to consensus sequence B, whereas primers REVF7830.1 and REVF7830.2 encode compensatory mutations in the 3′ region of the primer, indicated in bold. ···denotes deletions, -sequence identity, letters indicate alternative bases in the corresponding positions relative to consensus sequence B. Consensus sequences for common HIV clades as well as less frequent isolates are denoted in bold. Panel B. Schematic overview of the Rev RNA amplification strategy. Open bar denotes regions outside of open reading frame of interest, hatched bar denotes RNA region exon 2 Rev, grey bar represent DNA intermediate products durig amplification process. For details on primer design and amplification refer to Method section.

**Figure 2:**
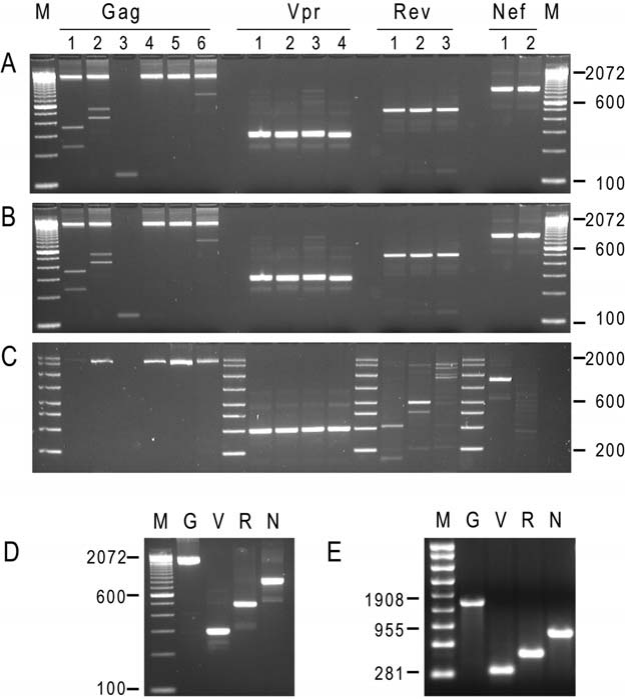
Successful clade-independent amplification of HIV RNA encoding for antigens from infectious plasma. Panel A: Agarose gel electrophoresis analysis of PCR fragment obtained from three diverse plasma. Amplification from subject plasma infected with Clade B sample. M: 100 bp DNA ladder (Invitrogen). Panel B: Amplification from subject plasma infected with Clade C virus. M: 100 bp DNA ladder (Invitrogen). Panel C: Amplification from subject plasma infected with Clade AG virus. M: AmpliSize DNA ladder (BioRad). Analysis of products obtained after the secondary PCR reaction for Gag, Vpr, Rev, and Nef as marked on the top. Panel D. cDNA obtained in preparative secondary PCR reaction corresponding to Gag, Vpr, Rev, and Nef antigens. M: 100 bp DNA ladder (Invitrogen). The molecular weight of representative DNA bands is indicated on the left. Panel E. RNA corresponding to Gag, Vpr, Rev, and Nef antigens obtained by *in vitro* transcription using amplified PCR products from subjects plasma. M: molecular weight RNA ladder (Promega), representative marker sizes are indicated on the left. G, V, R, N: in vitro transcribed RNAs for Gag, Vpr, Nef and Nef respectively.

Amplification of specific HIV genome regions is complicated by the high sequence diversity of the HIV genome. This sequence diversity prevents the design of a single universal primer pair for each gene of interest. To overcome this, we designed pools of forward and reverse primers for each target gene (i.e., Gag, Rev, Vpr and Nef) such that most virus strains will react with at least one forward and one reverse primer. Schematic representation of the primer design and strategy for the HIV RNA amplification are presented in **Figure 1** **.** This strategy provides for reliable amplification of intended target antigen genes, as well as the co-amplification of existing HIV quasispecies. A list of individual primers is given in **Table 1** and the composition of primer groups is given in **Table 2**. The number of amplification reactions for each HIV antigen was as follows: 6 for Gag, 4 for Vpr, 3 for Rev, and 2 for Nef. We amplified the four antigens from archived frozen plasma infected with diverse clades of HIV: B, C and AG (**Figure 2** **, panels A–C**). 2–3 mL of plasma were used to isolate HIV RNA and the titers of these three samples were of 53,334, 53,703 and 154,882 copies/mL, respectively. 2.5 µL of each eluted RNA was used in an RT-PCR for each antigen irrespectively of the initial viral load. PCR resulted in a productive amplification of DNA fragments of expected size for each antigen from all three samples.

**Table 1:** List of primers designed for amplification of HIV Gag, Rev, Vpr and Nef regions.

Primer Name	Primer Sequence
GAG F 124	ACTCTGGTAACTAGAGATCC
GAG F 304	AATTTTGACTAGCGGAGGC
GAG F 334	AGATGGGTGCGAGAGCGT
GAG F 334.1	AGATGGGTGCGAGACCGT
GAG R 1881	GCTCCTGTATCTAATAGAGC
GAG R 1881.1	GCTCCTGTATCTAATAAAGC
GAG R 1881.2	GCTCCTGTATCTAACAGAGC
GAG R 1913	TTTGGTTTCCATCTTCCTGG
GAG R 1913.1	TTTGGTTTCCATCTTCCTGC
GAG R 1913.2	TTTGGCTTCCATCTCCCTGG
GAG R 1913.4	TTTGGTTTCCATTTCCCTGG
GAG R 1913.5	TTTGGTTTCCATTTTCCTGG
VPR F 4995	GCAGGACATAACAAGGTAGG
VPR F 4995.4	GCAGGACATAACAAAGTAGA
VPR F 5058	AAGATAAAGCCACCTTTGCC
VPR R 5507	TTCTTCCTGCCATAGGAGATGC
VPR R 5507.1	TTCTTCCTGCCATAGGAAATGC
VPR R 5419	GCAGTTGTAGGCTGACTTCC
VPR R 5419.1	GCAGTTGTAGGCTGACTCCC
VPR R 5419.2	GCAGTTGTAGGCTGGCTTCC
REV F 7750	GGGATTTGGGGTTGCTCTGG
REV F 7750.1	GGGATTTGGGGCTGCTCTGG
REV F 7830	TGATAGTAGGAGGCTTGGTAGG
REV F 7830.1	TGATAGTAGGAGGCTTAATAGG
REV F 7830.2	TGATAGTAGGAGGCTTGATAGG
REV F 7911	GTTAGGCAGGGATATTCACC
REV F 7911.1	GTTAGGCAGGGATACTCACC
REV R 8300	CCCTGTCTTATTCTTCTAGG
REV R 8300.1	CCCTGTCTTATTCTTACAGG
REV R 8300.2	CCCTGTCTTATTCTTGTAGG
NEF F 8235	TAGCTGAGGGGACAGATAG
NEF F 8235.1	TAGCTGAGGGAACAGATAG
NEF F 8235.2	TAGCTGGCTGGACAGATAG
NEF F 8343	ATGGGTGGCAAGTGGTCAAAAAG
NEF F 8343.1	ATGGGTGGCAAGTGGTCAAAACG
NEF F 8343.2	ATGGGTGGCAAATGGTCAAAAAG
NEF F 8343.3	ATGGGTGGCAAGTGGTCAAAAGG
NEF R 9069	CCAGTACAGGCAAAAAGC
NEF R 9069.1	CAGTACAGGCGAAAAGC
NEF R 9069.2	CAGTACAGGCAAGAAGC

All sequences listed in 5′-3′ orientation. 5′ primers are represented by letter F (forward) and 3′ primers are represented by letter R (reverse). Numbers correspond to relative location of each primer within sequence chosen as the reference.

**Table 2:** Composition of primer groups.

GAG F 124	VPR F 4995	REV F 7750	NEF F 8235
GAG F 124	VPR F 4995	REV F 7750	NEF F 8235
**GAG F 304**	VPR F 4995.4	REV F 7750.1	NEF F 8235.1
GAG F 304	**VPR F 5058**	**REV F 7830**	NEF F 8235.2
**GAG F 334**	VPR F 5058	REV F 7830	**NEF F 8343**
GAG F 334	**VPR R 5507**	REV F 7830.1	NEF F 8343
GAG F 334.1	VPR R 5507	REV F 7830.2	NEF F 8343.1
**GAG R 1881**	VPR R 5507.1	**REV F 7911**	NEF F 8343.2
GAG R 1881	**VPR R 5419**	REV F 7911	NEF F 8343.3
GAG R 1881.1	VPR R 5419	REV F 7911.1	**NEF R 9069**
GAG R 1881.2	VPR R 5419.1	**REV R 8300**	NEF R 9069
**GAG R 1913**	VPR R 5419.2	REV R 8300	NEF R 9069.1
GAG R 1913		REV R 8300.1	NEF R 9069.2
GAG R 1913.1		REV R 8300.2	
GAG R 1913.2			
GAG R 1913.4			
GAG R 1913.5			

Primers were combined according to their position in the genome.

Bold indicates the primer group name. Non-bold: names of the primers which comprise each primer groups. Sequence of all primers is given in Table 1.

The example of purified products from preparative PCR is shown in **Figure 2** **, Panel D**. The reactions were set exactly as the secondary PCR reaction described in the Methods, but in identical replicates to generate sufficient mass of cDNA for the *in vitro* transcription reaction. Sequence analysis of these fragments confirmed that the amplified cDNAs correspond to Gag, Vpr, Rev and Nef. Products from the nested PCR reactions were transcribed *in vitro* to generate RNA and all four antigens were transcribed successfully (**Figure 2** **, Panel E**).

Because of the HIV genome diversity and presence of deletion and insertions within the open reading frames of interest, the molecular weight of cDNA is expected to vary. We performed a detailed analysis of cDNA and *in vitro*-transcribed RNA molecular weights for all four antigens amplified from 10 distinct infectious plasma samples. The size of the cDNA was measured by migration on an agarose gel relative to molecular weight markers. The observed size distribution of the cDNA analyzed by non-denaturing agarose gel electrophoresis was 1572±150 for Gag, 293±25 for Vpr; 496±25 and 841±50 for Nef. The observed size distribution for amplified RNA analyzed by denaturing agarose gel electrophoresis was 1761±54 for Gag, 338±37 for Vpr, 427±31 for Rev and 801±52 for Nef. The range of molecular weights for each antigen observed with these 10 samples is indicative of the high degree of subject-to-subject antigen sequence diversity.

Summary of samples from 33 patients with diverse viral load from which all four antigens were amplified is given on **Table 3**. Whenever possible, a greater volume of viral plasma was used for HIV RNA extraction to achieve higher yields of viral RNA. The sample with the lowest viral load examined was sample 1. For this sample the first cDNA synthesis reaction contained 2220 HIV RNA copies. Due to the multiplex design of the amplification, the RT reaction is divided into multiple PCR reactions. Since the Gag single strand cDNA is divided between 6 PCR reactions, the actual copy number in each PCR reaction is 370. A similar result was obtained for sample 3 with a higher viral load but smaller volume of available plasma. For this sample, each RT reaction contained 1580 copies of RNA and each PCR reaction for Gag contained 263 copies of cDNA. The calculation of final recovered HIV RNA concentration assumes no loss during the extraction procedure. With losses, the absolute copy requirement would be even lower. Overall these data demonstrate consistently successful amplification of all four antigens from plasma samples with diverse viral loads.

**Table 3:** List of samples tested for HIV RNA amplification.

sample number	viral load RNA/mL	volume of plasma used	clade	coies of RNA per RT reaction
1	7,413	3	B	2220
2	14,791	3	B	2220
3	15,849	2	C	1580
4	16,596	3	B	2480
5	18,197	3	B	5460
6	22,155	3	B	3320
7	22,909	2	AG	2300
8	28,840	3	B	8640
9	33,884	3	B	10160
10	38,663	3	B	5800
11	38,905	3	B	5840
12	45,709	3	B	6860
13	48,627	1	Nd	2440
14	50,070	1	Nd	2500
15	50,119	3	B	7520
16	50,119	3	B	7520
17	53,334	2	B	5340
18	53,703	2	C	5360
19	53,725	3	B	8060
20	72,865	1	Nd	3640
21	72,978	1	nd	3640
22	95,637	2	B	9560
23	117,490	3	B	17620
24	131,826	3	B	19780
25	134,000	3	B	20100
26	146,148	1	B	7300
27	154,882	3	AG	23240
28	158,489	3	B	23780
29	244,000	1	B	12200
30	513,000	3	Nd	38480
31	1,138,560	3	B	170780
32	1,479,108	3	Nd	221860
33	3,221,835	1	B	161100

Samples acquired as indicated in various geographical locations; nd: data not available; viral load test was estimated by approved clinical laboratory methods (Amplicor HIV assay Roche or bDNA Bayer); copies of RNA per RT reaction calculated assuming no loss of HIV RNA during isolation procedure. All samples obtained from randomly selected patients.

### Multiplex PCR method amplifies diverse HIV quasispecies

An advantage of this approach for antigen generation is its ability to capture HIV mutations which evolve under dynamic host CTL pressure [11]. It is broadly applicable to the general HIV-infected population irrespective of Clade designation, but also anticipates that it would capture various quasispecies present in a given subject. This is the cornerstone of our novel therapeutic paradigm which enables targeting, not only of dominant viruses, but also newly emerging virus populations which evolve as a result of immune pressure.

**Figure 3:**
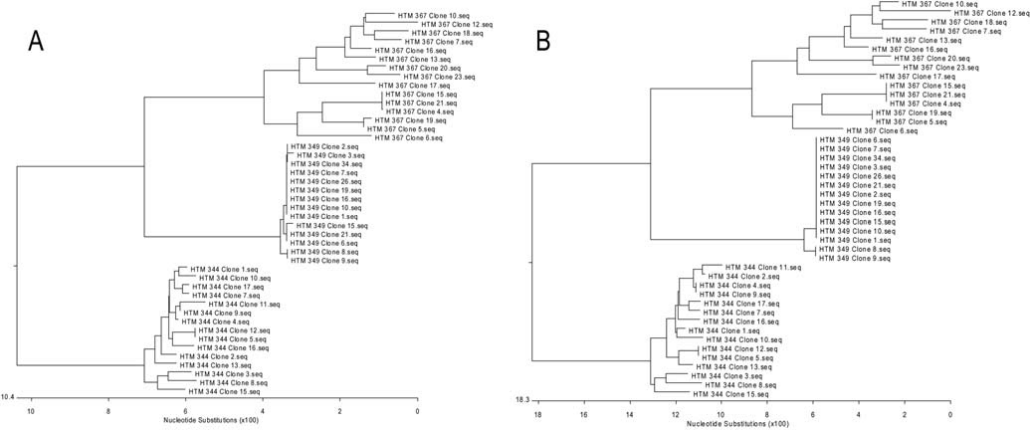
Capture of HIV quasispesies using the developed multiplex RT-PCR approach. Phylogenetic relationships of nucleotide sequences of isolated full-length Nef clones (Panel A) and amino acid sequences (Panel B). Horizontal scale indicates the number of nucleotide mutations or amino acid substitutions on each clone relative to neighbor clones.

To test our hypothesis that multiple quasispecies are co-amplified from a given subject, PCR fragments encoding full length Nef cDNA amplified from Clade B samples HTM-349, HTM-367 and HTM-344 (viral load 513,000; 53,334 and 95,637 copies per mL respectively) were cloned, sequenced and analyzed using phylogenetic tree analysis. A total of 15 clones were analyzed for each subject (**Figure 3** **)**. The analysis demonstrated that the cDNA population did indeed capture genes encoded by various HIV quasispecies. Phylogeneic tree analysis demonstrated that each subject's Nef sequences grouped within other sequences from that subject and were distinct from another subjects' sequences. More interestingly however is the observation that the number of the Nef variable sequences differed in each subject. At the nucleotide level (**Figure 3** **, Panel A**) the subject HTM 344 displayed greater diversity where out of 15 clones analyzed, 14 clones were unique, followed by subject HTM 367 with 13 unique clones and for subject HTM 349 only 6 unique clones. The subject-specific sequence clustering together with the variable number of unique clones between patients eliminates the possibility that the mutations are random mutational artifacts introduced during RT-PCR. Not every nucleotide mutation leads to an amino acid substitution, so the diversity is lower at the level of amino acid sequence (**Figure 3** **,** **Panel B**) with the same order of diversity trend for the three subjects. Similar analyses were performed for cDNAs encoding Gag, Rev and Vpr cDNA amplified from various subjects (data not shown). These data indicate that, as predicted, the multiplex RT-PCR is capable of capturing various quasispecies within each individual subject.

We next performed further analysis of the Nef sequences and scored individual primers on the level of productive secondary PCR reactions. Since the same formulation of primers was used with all samples independent of clade, we were interested to learn which primers within the formulated groups were leading to productive amplification and analysis of preferential use of forward and reverse primers was performed (**Table 4** **)**. The forward primer utilization of sample HTM 344 demonstrated that 12 out of 15 clones were formed by primer F8343.1, however in sample HTM 367 a different primer, F8343 formed the majority of clones. Interestingly, a novel primer annealing sequence was identified. The sequence was termed “new” and differed from either F 8343 or F8343.1 primer sequence by 2 nucleotides in the most 3′ position. We believe that the new sequence was formed due to the 3′ exonuclease proof-reading activity of the PCR enzyme. Similarly, different preferences for use of reverse primers were observed although no new reverse primers were identified in all three groups analyzed. The 3′ end of the PCR fragment is defined by the cDNA synthesis step during the RT reaction, and the lack of “repaired” primers is most likely due to lack of proofreading activity in the RT enzyme. Similar analyses were performed on multiple Rev clones and confirmed the original observation (data not shown). Since the sequence within regions of interest varies from patient to patient, the preferred utilization of the primers in the PCR reactions differ as well.

**Table 4:** Selective utilization of primers by RT-PCR from various subjects materials.

		Utilization in RT-PCR in various subjects		
Primer name	Primer sequence 5′-3′	HTM 344	HTM 349	HTM 367
T7 Nef F 8343	TAATACGACTCACTATAGGGAGACCACCATGGGTGGCAAGTGGTCAAAA**AG**	1	0	10
T7 Nef F 8343.1	TAATACGACTCACTATAGGGAGACCACCATGGGTGGCAAGTGGTCAAAA**CG**	12	0	0
New	TAATACGACTCACTATAGGGAGACCACCATGGGTGGCAAGTGGTCAAAA**GG**	2	15	5
	TAATACGACTCACTATAGGGAGACCACCATGGGTGGCAAGTGGTCAAAA**AT**			
64T Nef R 9069	(T)_64_CCAGTACAGGCAAAAAGC	7	8	11
64T Nef R9069.1	(T)_ 64_CAGTACAGGCGAAAAGC	6	2	4
64T Nef R9069.1	(T)_ 64_CCAGTACAGGCAAGAAGC	2	5	0

The Nef cDNA sequences were analyzed in the regions corresponding to the regions defined by the primers and identity of the primers was identifies by sequence. Total of 15 Nef clones were analyzed for subjects HTM344, HTM 349 and HTM 367. The number in the right three columns represents how many clones contained the identified primer.

### HIV RNA-transfected DCs stimulate antigen specific T cells in vitro

**Figure 4:**
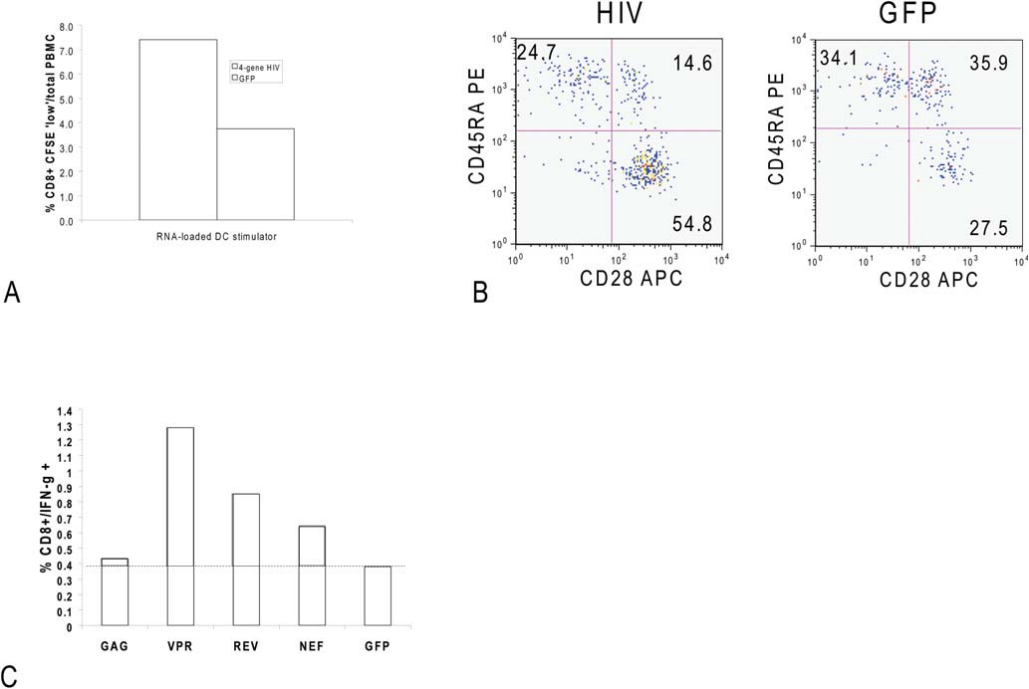
Panel A: CFSE-low cells expressed as a percentage of total PBMCs. Mature DCs (CD209: 96%; CD14: 0%; CD80: 100%; CD83: 91%; CD86: 100%; HLA-DR: 96%; and HLA-I: 100%) were electroporated with 4 HIV antigen-encoding RNAs (hatched bar) or eGFP (solid bar) were cultured with CFSE-labeled PBMCs for 6 days. Frequency of CD8+ CFSE-low were cells determined by flow cytometry. Panel B: CD28/CD45RA phenotype of CD8+ cells induced to proliferate (CFSE-low) by DC electroporated with 4 HIV antigen-encoding RNAs (left panel), as compared to the frequency of CD8+ CFSE-low cells induced by eGFP-RNA loaded control DC (right panel), as determined by flow cytometry. Panel C: Frequency of IFN-γ positive cells within the CD8+ CFSE-low subset induced by 4 hr re-stimulation with DC expressing individual HIV antigen-encoding RNAs, or eGFP control RNA, as determined by intracellular staining and flow cytometry. The background response for single HIV RNA stimulators (1ug HIV RNA/10^6^ DC) was calculated at 0.38% from GFP RNA-electroporated DC (1ug GFP RNA/10^6^ DC) and is indicated by the horizontal dashed line.

The goal of active HIV immunotherapy is to stimulate the preferential expansion of antiviral effector T cells. To demonstrate that HIV RNAs generated by our approach can express antigens capable of inducing CD8+ T-cell immunity, we prepared DC electroporated with all four autologous HIV antigens encoded as RNAs. 1 µg Gag RNA, 0.25 µg Nef RNA, 1 µg Rev RNA, and 1 µg Vpr RNA were electroporated along with 1 µg of CD40L RNA per 10^6^ DC. Since cells were fully matured by overnight incubation in the presence of TNFα, INFγ and PGE_2_ the maturation status of the DCs did not change after electroporation with the RNAs (Data not shown). These cells were co-cultured with autologous PBMCs pre-labeled with CFSE. After 6 days of co-culture, the frequency and phenotype of proliferating cells was detected by residual CFSE fluorescence within the CD8+ T cell population with effector (CD45RA+/CD28−) or effector/memory (CD45RA−/CD28+) phenotypes. After 6 days of co-culture, the CD8+ T-cell population was stimulated with either eGFP RNA-transfected DC (negative control) or HIV RNA-transfected DC. The frequency of CFSE-low cells stimulated with GFP RNA-loaded DC was 3.75% while those stimulated with HIV RNA-loaded DC had a frequency of 7.41% (**Figure 4** **Panel A**). No proliferation above the negative control background was observed within the CD4+ T cell subset, with all DC populations inducing ∼1% CD4+ CFSE-low cells within total PBMCs (data not shown). Within the proliferating CFSE-low CD8+ T cell subset stimulated with HIV RNA-loaded DC, 24.7% of cells exhibited a phenotype consistent with fully differentiated effector cells (CD8+CD28−CD45RA+) versus 54.8% of cells had a phenotype indicative of effector/memory cells (CD8+CD28+CD45RA−) (**Figure 4** **Panel B**).

To assess the specificity and effector function of the T cells responding with antigen-induced proliferation, the cultures were further re-stimulated with either DC electroporated with GFP RNA (negative control), a pool of all 4 HIV RNAs, or each HIV RNA independently. After 4 hours CFSE-low CD8+ T cells were tested for IFN-γ expression by intracellular staining. Induction of IFN-γ expression above the GFP negative control background was observed for all HIV RNA DC-stimulated cultures (**Figure 4** **, Panel C**). Co-cultures that were originally established with GFP RNA-electroporated control DC for 6 days and then restimulated with individual HIV antigen-encoding RNA-electroporated DC all expressed less than 0.15% IFN-γ activity within the CD8+ CFSE-low subset (data not shown).

## Discussion

The strategy of the HIV therapy using autologous DC loaded with autologous antigen has been successfully tested in two clinical trials [14], [15]. The HIV therapy described here exploits similar therapeutic principles of the autologous approach but with added advantages in terms of potential safety and applicability to a broader HIV-infected patient population.

Our approach requires only a small amount of patient plasma, which may be pivotal for eventual commercial-scale manufacturing. We demonstrated amplification from samples containing as little as 263 or 370 copies of cDNA in a single PCR reaction. Many RT-PCR methods such as HIV sequence detection methods detect as low as 50 copies or even a single copy [22]–[24]. However, such methods developed for high sensitivity detect only small amplicons and do not require an intact HIV genome. To clone longer HIV fragments, 1,000 copies of the HIV genome are required for use in the initial RT reaction [25]. Therefore, the HIV RNA copy requirements described here is comparable to those reported in the literature. Due to the fact that RNA is amplified, first through RT-PCR, and then by *in vitro* transcription, milligram-scale RNA masses can be achieved, sufficient to transfect large numbers of autologous DCs.

The complete coding regions for p55 Gag and Nef and partial products for Rev and Vpr were amplified. The full length Rev mRNA is formed in the course of a trans-splicing reaction which is not possible to reproduce *in vitro*. Since the exon 1 of Rev encodes only 25 amino acids we designed primers to amplify exon 2 only. The ATG initiation codon which enables translation of that RNA is introduced in the context of a secondary PCR primer. In the case of Vpr, primers were designed to amplify the sequence coding for amino acids 1–71 only. All of the predicted epitopes for CTL recognition within the Vpr protein are located between amino acids 12–70 (http://www.hiv.lanl.gov/content/immunology). The approach of expressing functionally inactive HIV genes which are still able to successfully induce immunity have been documented by others [26].

Our ability to amplify HIV sequences in a clade-independent manner rests on the principles of multiplex RT-PCR technology. First, different loci within HIV genomes are targeted in primary PCR reactions and secondly, each primer group is composed of primers which are complementary to target sequences as well as additional primers carrying compensatory mutations. During the annealing step the most complementary primer-template combination gives rise to a primary product which is then amplified further in a secondary PCR reaction. We believe that because the template is first amplified in a course of primary PCR, mismatches between primary PCR fragments and secondary PCR primers is more forgiving and allows for productive amplification. Therefore greater numbers and combinations are required for the primary PCR reactions. This study demonstrates the feasibility of target region amplification from infectious plasma encompassing various HIV virus clades as well as diverse viral loads. We also demonstrate that this process captures various quasispecies within a single patient. We also observed that the diversity of Nef sequences found in subject HTM-349 is dramatically reduced compared to the other two subjects analyzed. It would be interesting to determine whether the low diversity of Nef sequences is due to a lack of Nef-specific CTLs within that patient consistent with the hypothesis that immune pressure drives the evolution of Nef variants. We believe that the ability to capture autologous sequences of HIV virus and all quasispecies arising as a result of the CTL pressure is the foundation for the successful anti HIV immunotherapy.

While translated protein from the RNAs encoding the four individual antigens was studied in the DC, we were unable to devise a universal method of detection or identify an antibody which could cross-react with all subject-specific amplified material. Also, the sensitivity of these methods is insufficient to detect protein expression when a relatively low amount of RNA (1 µg or less) is delivered to the DCs. Instead we elected to study presentation of antigens by RNA-electroporated DC with detection of induced T-cell responses as a surrogate assay for protein expression using PBMCs derived from a successfully HAART-treated HIV-infected donor. Autologous RNA rather than consensus-sequence- based reagents (vectors, peptides, etc.) was used to transfect DCs to maximize the probability of antigen recognition

Flow cytometric analysis of T cell- DC co-cultures demonstrated that DCs electroporated with the four HIV antigen-encoding RNAs successfully induced specific proliferation and effector function (IFN-γ activity) to all four antigens within the responder CD8+ T cell subset in this experiment. Both qualitative and quantitative issues contribute to the reported immune responses. The RNAs used in this experiment have different molecular weights: 1.5 kb for Gag and 0.3 kb for Vpr thus the number of molecules of each RNA is inversely proportional to the mass used. Under the conditions used in the experiment shown in Figure 4, the relative number of molecules delivered to the DC is greater for Vpr and lower for Gag. Figure 4C shows the correlation of antigen reactivities (as measured by IFN-γ release) supporting the idea that for this donor, the relative epitope densities presented on the surface of the DC is proportional to the number of RNA transcripts electroporated for each antigen.

The preferential targeting of Vpr with CTLs was also reported by others [20]. However, it is well documented that different patients can have qualitatively different preferential epitopes reactivities resulting in non-linear relationships with antigen expression levels or epitopes densities on antigen presenting cells. This relationship is further impacted by differences in TCR avidity for specific epitopes or proteosome processing preferences which can vary from patient to patient and whether the predominant infecting virus species encode mutated epitopes [27]. A study of monozygotic twins infected at the same time with the same virus demonstrated that while some CD8 T cell specific responses were the same, some discordinant T cell responses were also found [28]. The discordinant responses cannot be simply explained by virus phenotype or HLA epitope restriction and suggests that additional factors play a role in selection of epitope recognition.

We also noted that the majority of the cells responding to HIV antigens with proliferation and IFN-γ production exhibited a CD28+/CD45RA- ‘effector/memory’ phenotype which has been linked to long-term non-progression [5], [6]. Collectively, these data provide strong evidence that, at least *in vitro*, HIV antigens encoded by RNA can be translated and presented by DC to induce poly-antigen immunity. Such activity is presumed to be an essential aspect of an immunotherapeutic designed to control HIV viral escape. In addition, no specific activity was recorded within the CD4^+^ subset, consistent with the inability of antigen encoded by RNA to efficiently target the endosomal pathway [29]. We speculate that there may be specific advantages to being able to induce antiviral CD8+ T cell immunity without concomitant expansion of CD4+ T cells which might serve as a reservoir for virus and facilitate enhanced viremia.

The breadth of the CTL repertoire has been shown to correlate with anti-HIV-protective immunity. In an animal model comparing immune responses in groups of animals immunized with either a gag-pol-encoding DNA vector, codon optimized Gag-Pol, or multivalent Expression Library Immunization (ELI) therapeutics, the induced immune responses were up to 10-fold higher in the group immunized with the multivalent (ELI) therapeutic than the other two constructs tested [30]. Also, improved protection against simian AIDS was reported in a study with an experimental immunotherapy directed against six antigens (Gag, Pol, Env, Rev, Tat, Nef) compared to one directed against only three antigens (Gag, Pol, and Env) [21]. In addition, immunotherapies targeting multiple regions of the viral genome have the potential to force the accumulation of multiple mutations as a consequence of polyvalent CTL pressure which can drive the virus into a state of poor replicative fitness [31].

This study establishes the feasibility of multiplex RT-PCR-mediated amplification of autologous RNA encoding HIV antigens from small volumes of infectious plasma. We also demonstrate that this approach captures multiple quasispecies present within a given subject which could allow the induction of multivalent CTL responses, a critical factor in minimizing the chances of rapid emergence of viral escape variants. Furthermore, we demonstrate ability of DCs transfected with autologous amplified HIV antigen RNA to induce antigen-specific CD8+ T cells.

## Methods

### Design of primers for strain-independent amplification of HIV antigens

The strategy used to design primer pools for PCR amplification for the selected HIV antigens, using the Rev sequence as an example, is summarized in **Figure 1**, Sequences of HIV isolates were aligned using BLAST analysis with sequence NC_001802 serving as an arbitrary reference [32], [33]. Nucleotide regions which appeared to have sequence conservation were selected for primer design. The strategy used to design primers for PCR amplification, using Rev sequence as an example, is summarized in **Figure 1**. As shown a primer that is complimentary to the consensus B sequence was designed (Rev F 7830). Alternative primers which could also accommodate the frequently found mutations in this location were designed as well. These alternative primers contain compensatory sequence variations to accommodate the frequently found mutations in positions 7847 and 7848 (Rev F 7830.1 and Rev F 7830.2).

To minimize the number of primers used, a mismatch at the 5′ end of a primer sequence was tolerated since lowering the PCR amplification stringency could compensate for such mismatches. However, mismatches at the 3′ end of a primer sequence were avoided

To enable transcription of the PCR product *in vitro,* the products of the primary PCR reaction were modified to insert a T7 RNA polymerase binding site at the 5′ end (**Figure 1**). Naturally occurring translation initiation codons for Gag, Vpr and Nef were captured during PCR amplification. However Rev mRNA is formed in a transplicing event and capture of a full length cDNA via PCR is not achievable. Only the second exon of Rev is amplified, so the addition of the initiator ATG codon for the Rev antigen in a nested round of PCR is required in order to enable translation initiation. The reverse primers contain a poly(T)_64_ tail which is transcribed into a poly(A)_64_ tail on the synthesized RNAs. (**Figure 1**). Individual primer sequences for the primary round of amplification are provided in **Table 1** **.**

### Formulation of primer groups

Oligonucleotides (IDT) were reconstituted at a concentration of 100 mM. Primers were combined into groups to reduce the number of PCR reactions (the composition of primer groups is provided in **Table 2**. The final primer concentration in formulated stock solutions was 5 µΜ for PCR, and 20 µΜ for gene-specific reverse transcription. The amplification protocol was simplified by grouping primers according to their location. The number of amplification reactions for each HIV antigen was significantly reduced from the scenario where individual primer combinations would be used: 6 for Gag, 4 for Vpr, 3 for Rev, and 2 for Nef. Once primer mixes were made they were not further changed and the same formulations of primers were used to amplify various plasma materials.

### Isolation and amplification of HIV antigens from patient plasma

HIV RNA was isolated from 1 to 3 mL of plasma from HIV patients using a NucliSens kit (BioMerieux), according to the manufacturer's instructions and eluted in 30 µL of nuclease free water. First strand cDNA synthesis reaction contained gene-specific primers for either Gag, Vpr or Rev, and oligo dT_(20)_ (Invitrogen) for Nef, 40 units of RNAseOut (Invitrogen), 0.5 mM of each dNTP (Clontech), and Superscript first strand buffer. After annealing at 65°C for 5 minutes, DTT to 5 mM and 400 units of Superscript III (Invitrogen) were added and the reaction was incubated at 55°C for 1 hour.

2.5 µL of the first strand cDNA reaction was then taken into a primary PCR reaction containing 5 units of PFU ultra HS, PFU buffer (Stratagene), 0.2 mM of each dNTP (Stratagene), and the corresponding group of primers at a final concentration 0.4 µM for Gag, 0.6 µM for Vpr, 0.2 µM for Rev, and 0.4 µM for Nef, in a final reaction volume of 50 µL. The PCR reaction was denatured at 95°C for 2 minutes and then run for 40 cycles as follows: 95°C for 30 seconds, 54°C for 30 seconds, and 72°C for 3 minutes and 10 minutes for the last cycle. The annealing temperature was kept at 54°C here and in the secondary PCR amplification to allow for annealing of primers to templates with a limited degree of mismatch [34]

1 µL of the primary PCR reaction was then taken into a secondary PCR reaction containing 2.5 units of PFU Ultra HS, PFU buffer (Stratagene), 0.2 mM of each dNTP (Stratagene), and gene specific T7 and 64T groups of primers, in a final reaction volume of 25 µL. The cycling parameters were the same as in the primary PCR reaction. Products of the secondary PCR reaction were purified using a QIAquick purification column (QIAGEN). For preparative PCR several (i.e. 6 or 12) reactions were established with same conditions as secondary PCR except the total volume of each reaction was 50 µL.

### PCR Amplification of HIV sequences from noninfectious templates

Gag, Rev and Vpr were amplified from plasmid pBKBH10S and Nef was amplified from plasmid p93TH253.3 obtained from NIH AIDS Research & Reference Reagent program [35], [36]. Single forward and reverse PCR primers were selected with full complementarity to the template determined by sequence analysis. All PCR conditions were exactly the same as used for amplification of the infectious material.

### *In vitro* transcription of HIV antigens

Secondary PCR fragments served as templates for an *in vitro* transcription reaction using mMessage mMachine T7 Ultra kit (Ambion) according to the manufacturer's instructions. The amplified RNA was purified using RNeasy columns (QIAGEN).

### Sequence analysis and phylogeneic relationship

Sequencing analyses were performed using the UNC sequencing facility (University of North Carolina, Chapel Hill). Nucleotide sequence analysis, identity verification and phylogeneic tree analysis were performed using Lasergene software (DNAStar), the Los Alamos HIV Sequence Database (http://www.hiv.lanl.gov), and BLAST analysis [32], [33].

### Isolation of human dendritic cells

A leukapheresis sample from a volunteer was collected on a COBE Spectra (Gambro BCT) using the AutoPBSC procedure described by Lifeblood (Memphis, TN). Peripheral blood mononuclear cells were isolated using a Ficoll density gradient (Histopaque®-1007 Hybri-Max®, Sigma) and cultured for 1 to 2 hours to allow adherence of the monocytes. Non-adherent cells were removed and the remaining monocytes were cultured in X-VIVO 15™ (Cambrex) medium for 6 to 7 days, supplemented with 1000 U/mL each of GM-CSF (Berlex, Leukine® liquid) and IL-4 (R&D Systems).

### Generation of DC for functional testing of HIV IVT RNAs for the induction of anti-HIV immunity *in vitro*

Immature DCs were generated as described above from a successfully HAART-treated HIV donor with a viral plasma copy number of less than 200 copies per mL. To achieve DC maturation, immature DC were cultured on day 5 with 10 ng/ml TNF-α, 1000 µg/ml IFN-γ, 1 µg/ml PGE_2._ On Day 6, matured DCs were co-electroporated with *in vitro* transcribed RNA encoding CD40L at 1 µg per million of DC, and 1 µg Gag, Rev, Vpr and 0.25 µg Nef autologous HIV RNAs per million of DCs. A negative control DC stimulator was generated by transfecting DC with CD40L RNA and 3.25 µg eGFP RNA, instead of HIV RNA mix. RNA-electroporated DC were further cultured for 4 hrs in X-VIVO-15 medium without additional cytokine supplements.

### *In vitro* co-culture of DC and PBMC from an HIV-infected subject to induce anti-HIV T-cell responses to multiple HIV antigens

#### CFSE labeling

PBMCs from the HIV donor were enriched by Ficoll gradient separation, washed twice with PBS and re-suspended at 2.0×10^7^ cells per mL in PBS. CFSE was added to the cell suspension for a final working concentration of 1.0 µM and incubation for 8 minutes at room temperature. The staining was quenched by the addition of an equal volume of Human AB Serum and incubation for 2 minutes.

#### Initial DC/PBMC co-culture

Cultures of HIV RNA-electroporated mature DC, and eGFP-RNA control DC were established in parallel with CFSE-labeled PBMC at a 1∶10 ratio, 1 million total cells/mL in 5% Human AB serum for 6 days at 37°C, 5% CO_2_.

#### Cell surface phenotyping of proliferating CFSE ‘low’ labeled cells

After 6 days of culture, PBMCs were harvested, washed once with 2 mL PBS containing 10% FBS and stained for surface antigens using CD45RA PE, CD8 PerCP-Cy5.5, CD28 APC or CD45RA PE, CD4 PerCP-Cy5.5, CD28 APC antibodies (BD Bioscience) at room temperature in the dark for 20 minutes. Samples were washed twice with cold PBS containing 10% FBS and re-suspended in 300 µL of 2% BD Cytofix (BD Bioscience) Samples were acquired on a BD FACSCalibur flow cytometer and analysed using FlowJo software (Three Star, Inc.) Analysis gates were set on the basis of FSC v. SSC to define viable lymphocytes and lymphoblasts, and the frequency of proliferating cells determined by detection of CFSE ‘low’ cells, and their associated cell surface phenotype

#### Measurement of anti-HIV specific activity by restimulation of PBMCs with individual DC populations expressing a single HIV gene

Immature DC were generated as described above, matured with TNF-α, IFN-γ and PGE_2_ and cells split into 5 groups, allowing for DC populations to be generated expressing just a single HIV gene from the panel of four individual antigens, and a fifth DC population electroporated with eGFP RNA, as negative control. The DC populations were co-cultured in parallel with CFSE-labeled PBMC harvested from the previous 6-day co-culture described above. One hour after re-stimulation with DCs, 0.25 µl of Golgi plug (BD Bioscience) was added to each sample and incubated for an additional 3 hours at 37°C, 5% CO_2_ in RPMI containing 10% Human Serum. Samples were washed once with 1 ml PBS containing 10% FBS and stained for surface antigens using CD8 PerCP-Cy5.5 or CD4 PerCP-Cy5.5 antibodies at 4°C in the dark for 20 minutes. After wash with PBS containing 2% FBS and re-suspension in 150 µl of 2% BD Cytofix cells were incubated at room temperature in the dark for 20 minutes. Samples were washed twice with 1 ml of Perm/Wash buffer (BD Bioscience) and incubated at room temperature in the dark for 20 minutes with 2 µl of purified Mouse IgG_1_ antibody. Samples were stained for intra-cellular cytokines using IL-2 PE and IFN-γ APC antibodies at room temperature in the dark for 20 minutes. Samples were washed twice with 1 ml of BD Perm/Wash buffer and re-suspended in 150 µl of 2% BD Cytofix, acquired on a BD FACSCalibur flow cytometer and analyzed using FlowJo software. PBMC that had proliferated (CFSE ‘low’) during the previous 6-day co-culture were gated and analyzed for induced IFN-γ and IL-2 content.
